# Chemotherapy-Free Polatuzumab Vedotin and Rituximab (Pola-R) for Frail, Older Patients with Relapsed DLBCL: A Case for Geriatric Assessment and Shared Decision-Making

**DOI:** 10.3390/reports9030318

**Published:** 2026-09-21

**Authors:** Ryusuke Horaguchi, Shohei Kikuchi, Kento Ono, Tomoki Minemura, Tsutomu Sato

**Affiliations:** Department of Hematology, Faculty of Medicine, Academic Assembly, University of Toyama, 2630 Sugitani, Toyama 930-0194, Japan

**Keywords:** diffuse large B-cell lymphoma, frailty assessment, geriatric assessment, chemotherapy-free regimens, polatuzumab vedotin

## Abstract

**Background and Clinical Significance:** Older, frail patients with relapsed diffuse large B-cell lymphoma (DLBCL) face limited treatment options due to poor tolerance for standard cytotoxic chemotherapy. While novel chemotherapy-free regimens like polatuzumab vedotin plus rituximab (Pola-R) have emerged, optimizing their use requires careful evaluation of patient vulnerability via geriatric assessment (GA) and shared decision-making (SDM). **Case Presentation:** We present the case of a male in his late 70s with multiple-relapse DLBCL who was classified as frail by a comprehensive geriatric assessment, complicated by cognitive impairment and functional decline. Due to his frailty and previous intolerance to cytotoxic chemotherapy, a safety-prioritized, chemotherapy-free Pola-R regimen was selected through an SDM process in which his family supported his decision-making. The patient achieved a complete metabolic response without severe adverse events and has remained progression-free for 15 months. **Conclusions:** This case illustrates the feasibility and potential clinical activity of the chemotherapy-free Pola-R regimen for frail older patients with DLBCL, underscoring the critical role of integrating GA and SDM to individualize therapy and honor patient goals.

## 1. Introduction and Clinical Significance

Older, frail patients with diffuse large B-cell lymphoma (DLBCL) face inferior clinical outcomes compared to younger, fit cohorts. Standard cytotoxic chemotherapy is often poorly tolerated, and optimal treatment strategies remain poorly defined, largely due to the underrepresentation of this population in clinical trials. In this context, the advent of novel agents, such as antibody–drug conjugates (ADCs) and bispecific antibodies (BsAbs), has enabled chemotherapy-free approaches, significantly expanding treatment possibilities for vulnerable older adults [1,2,3].

Because older adults represent a highly heterogeneous population, simply expanding treatment options is insufficient; clinical decisions must account for individual vulnerability and physiologic reserve. Relying solely on chronological age risks either increasing adverse events through over-treatment or inappropriately depriving patients of viable therapeutic opportunities. Therefore, comprehensive evaluation via a geriatric assessment (GA)—which systematically evaluates essential domains including physical and cognitive function, emotional health, comorbidities, polypharmacy, nutrition, and social support—is highly recommended [4,5,6]. While the simplified geriatric assessment (sGA) is a validated prognostic tool in malignant lymphoma [7,8,9], its routine clinical implementation and direct application to treatment optimization remain scarce. Combining GA-based individualized evaluation with novel chemotherapy-free agents holds great potential to optimize treatment for older patients.

Alongside evolving therapies, shared decision-making (SDM) has become critical in geriatric oncology, particularly when evidence-based recommendations must be individualized according to patient frailty, cognitive function, autonomy and personal values [10,11]. Here, we report the case of a multiple-relapse DLBCL patient identified as frail by GA, who safely achieved a complete remission using a chemotherapy-free polatuzumab vedotin plus rituximab (Pola-R) following an SDM process.

## 2. Case Presentation

**Figure 1:**
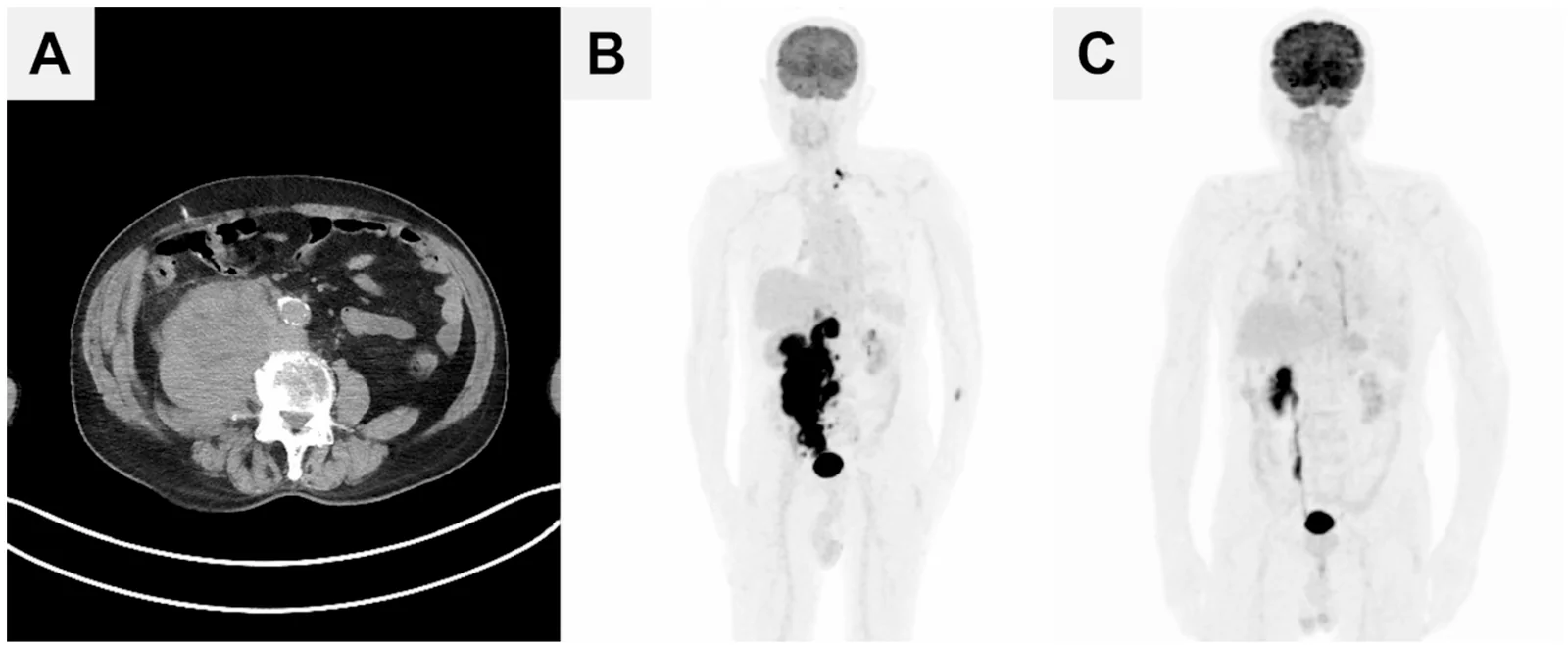
Axial non-contrast computed tomography (CT) imaging reveals a bulky psoas muscle tumor at the time of first relapse (**A**). 18F-fluorodeoxyglucose positron emission tomography (18F-FDG-PET) demonstrates intense FDG uptake (SUVmax: 34.63) within the lesion (**B**). Following four cycles of dose-reduced Pola-R-CHP and subsequent radiotherapy, maximum intensity projection (MIP) imaging of FDG-PET shows complete resolution of the FDG-avid mass, indicating a complete metabolic response (**C**).

A male patient in his late 70s with a history of primary central nervous system DLBCL (PCNSL)—in remission for 7 years after high-dose methotrexate and whole-brain radiotherapy—presented with a bulky retroperitoneal mass causing right hydronephrosis (Figure 1A,B). Biopsy confirmed DLBCL with positivity for CD20, BCL2, BCL6 and MUM1, and weak positivity for CD10. Given the history of PCNSL, the systemic lesion was clinically considered an extra-CNS relapse. However, because the original PCNSL was diagnosed primarily by cerebrospinal fluid cytology, pathological comparison and molecular clonality analysis were not performed, and de novo systemic DLBCL could not be excluded. The patient had bulky stage II disease and an International Prognostic Index (IPI) score of 3 (age > 60 years, performance status (PS) of 2, and elevated LDH), indicating high–intermediate risk.

On admission, a comprehensive geriatric assessment (CGA) was performed. Key findings included a G8 score of 14/17, with deductions primarily for cognitive impairment and mobility, while his body mass index (≥23) and nutritional status were well preserved (Table 1). He exhibited mild-to-moderate cognitive impairment, with a positive Mini-Cog result (clock drawing test score, 0; delayed recall score, 1; total score, 1/5) and a Mini-Mental State Examination (MMSE) score of 22/30, likely secondary to prior brain irradiation and hydrocephalus. He also showed moderate functional decline in activities of daily living. He was able to ambulate independently but exhibited unsteadiness—presumed to be due to impaired balance—and had a history of falls. He had a Katz ADL score of 2/6 (independent only in feeding and continence), and a Lawton IADL score of 2/8. While his Charlson Comorbidity Index was 1 (accounting for cognitive impairment), the CIRS-G identified scores ≥ 2 across five organ domains. He lived with his family and had adequate social support. Based on these findings, the simplified geriatric assessment (sGA) classified him as frail. Combined with an IPI score of 3, the Elderly Prognostic Index (EPI) categorized him as high risk.

Standard cytotoxic chemotherapy was deemed excessively risky due to his frailty. He was initiated on dose-reduced Pola-R-CHP (cyclophosphamide and doxorubicin at 70%). Supported by G-CSF, neutropenia was limited to grade 1/2, with no grade ≥ 3 adverse events observed. Despite achieving an interim response, he developed *Pneumocystis jirovecii* pneumonia (PJP) after four cycles. Primary PJP prophylaxis had not been initiated, and grade 3/4 lymphopenia was not observed. The PJP resolved with appropriate therapy, but cytotoxic chemotherapy was discontinued due to tolerability concerns. Radiotherapy (30 Gy in 15 fractions) was administered to the residual lesions, resulting in a complete metabolic response (CMR) on 18F-fluorodeoxyglucose positron emission tomography/computed tomography (FDG-PET/CT) (Figure 1C).

**Figure 2:**
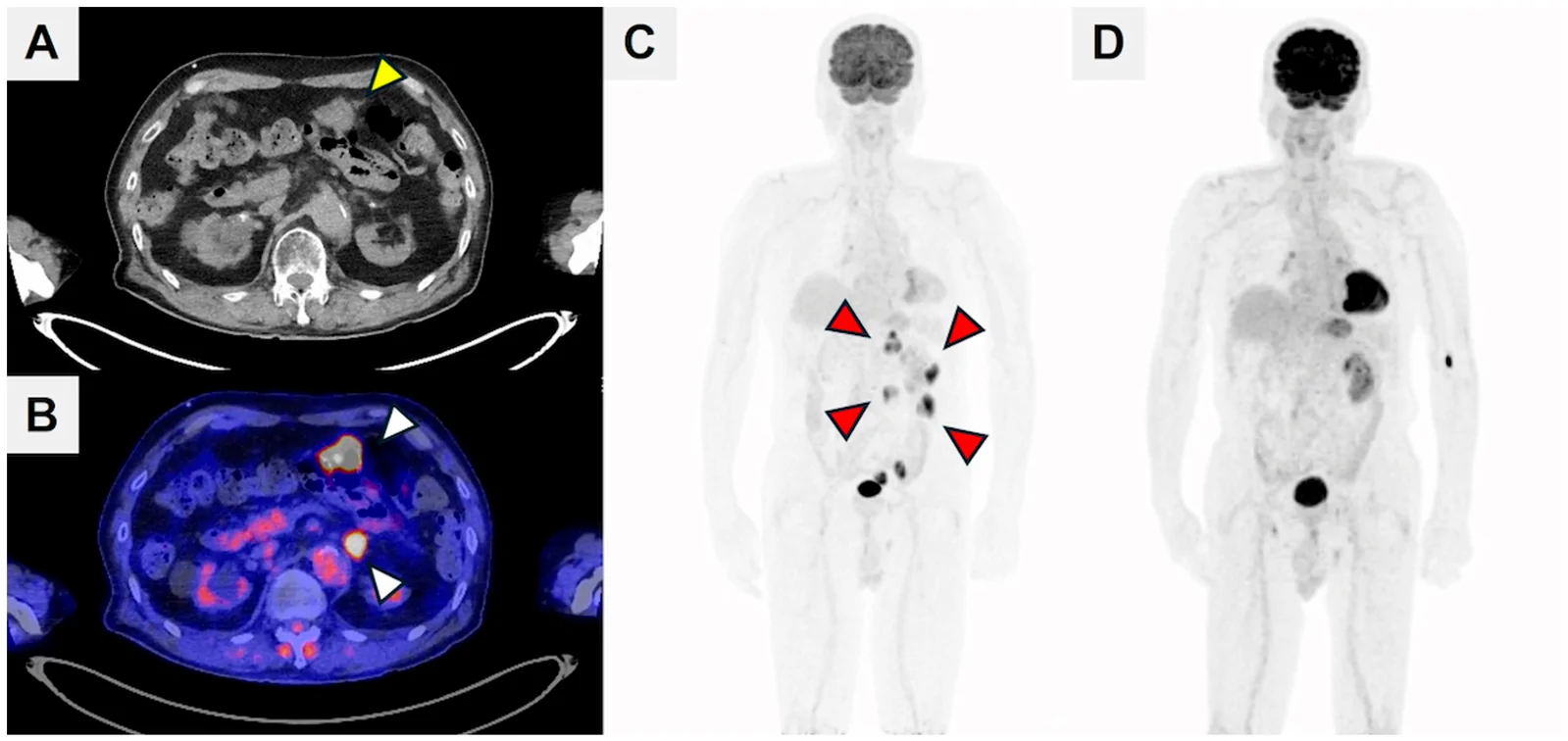
Multiple intra-abdominal masses are identified on CT imaging (yellow arrow, (**A**)) and FDG-PET/CT (SUVmax: 21.76 in the superior lesion and 22.31 in the posterior lesion; white arrows, (**B**)). MIP imaging highlights multiple FDG-avid lesions at the time of second relapse (red arrows, (**C**)), which completely resolved after six cycles of Pola-R therapy (**D**).

One year after the initiation of Pola-R-CHP, an intra-abdominal relapse with multiple nodal lesions occurred (Figure 2A–C). Although a repeat biopsy was considered, it was deferred because the lesions were located deep within the intra-abdominal cavity, posing substantial procedural risks. A repeat CGA revealed a one-point decline in his G8 score to 13/17, with other domains remaining stable. Given his frailty and prior history of PJP, the clinical team proposed best supportive care. However, the patient consistently expressed a strong preference for active treatment, although his understanding of chemotherapy-related risks appeared limited. His family was therefore engaged to support his decision-making. Through an SDM process that transparently weighed the prohibitive risks of conventional cytotoxic chemotherapy against the patient’s goals, a safety-prioritized, chemotherapy-free Pola-R regimen (polatuzumab vedotin 1.8 mg/kg and rituximab 375 mg/m^2^ every 3 weeks) was selected. The pola-R was initiated 11 months after the last dose of polatuzumab vedotin in the initial Pola-R-CHP treatment. The patient subsequently received six cycles of Pola-R without dose reduction, resulting in a total exposure of ten polatuzumab-containing cycles across both treatment periods (cumulative polatuzumab vedotin dose: 18.0 mg/kg).

Throughout and following the Pola-R treatment, PJP prophylaxis with atovaquone was maintained. Grade 3/4 lymphopenia was not observed, and the treatment was well tolerated without infectious complications, grade ≥ 3 hematologic toxicity, or peripheral neuropathy. After six cycles, he achieved CMR on FDG-PET-CT (Figure 2D) and has remained progression-free for 15 months, calculated from the initiation of Pola-R therapy.

## 3. Discussion

This case illustrates the feasibility and potential clinical activity of a chemotherapy-free regimen (polatuzumab vedotin plus rituximab) to achieve durable complete remission in a multiply relapsed, frail patient with DLBCL.

Therapeutic options for frail older patients with relapsed/refractory (R/R) DLBCL who cannot tolerate standard salvage chemotherapy are severely limited. At the time of this patient’s second relapse, therapeutic regimens approved under Japanese national health insurance were limited to polatuzumab vedotin plus bendamustine and rituximab (Pola-BR) and epcoritamab monotherapy; other novel regimens, such as tafasitamab and mosunetuzumab-containing regimens, were not yet approved, and glofitamab-containing regimens remain unapproved in Japan. While Pola-BR is established for transplant-ineligible disease [12], bendamustine-associated immunosuppression and infectious complications pose significant risks in frail individuals, particularly in our patient with a history of severe PJP during previous therapy. Furthermore, although epcoritamab monotherapy was an available chemo-free option, the potential risks of cytokine release syndrome and neurotoxicity—which could complicate or worsen his preexisting cognitive impairment and necessitate intensive inpatient management—raised safety concerns. Historically, rituximab monotherapy (R-mono) has been used in this setting, but its efficacy is insufficient. Achieving durable disease control remains challenging, yielding an overall response rate of 31–37% and a median progression-free survival (mPFS) of 1.7 months [13,14]. Consequently, Pola-R was justified as the most viable regimen balancing disease control, safety, and caregiver burden.

Polatuzumab vedotin provides a targeted, chemotherapy-free alternative. In a phase II R/R DLBCL study, Pola-R demonstrated an ORR of 50%, a CR of 35%, and an mPFS of 6.4 months (with a median duration of response of 11.3 months), suggesting superior durability compared to R-mono [15]. As molecular cell-of-origin and genomic analyses of the systemic lesion were not performed in this case, no association between the activated B-cell–like (ABC) subtype and the response to Pola-R could be established. Previous studies have reported that polatuzumab vedotin has activity in the ABC subtype [16], that the ABC subtype is more prevalent among older patients [1], and that the majority of PCNSLs are classified as the ABC subtype [17,18]. Regarding polatuzumab vedotin retreatment, clinical evidence remains lacking. Nevertheless, retreatment was considered reasonable because the initial Pola-R-CHP had been discontinued due to intolerance rather than disease refractoriness, and 11 months had elapsed since the last polatuzumab dose.

Looking forward, bispecific antibodies are further expanding chemotherapy-free options. The CD20/CD3 bispecific mosunetuzumab combined with polatuzumab vedotin (Mosun-Pola) demonstrated exceptional efficacy in phase III SUNMO trials (ORR 70.3%, CR 51.4%, mPFS 11.5 months) for transplant-ineligible R/R DLBCL [19]. Crucially, Mosun-Pola showed favorable safety in SUNMO, with low treatment discontinuation (2.2%) and manageable, low-grade cytokine release syndrome [19]. While formal GA was not utilized in SUNMO, this tolerability profile suggests Mosun-Pola could be highly relevant for frail older patients, warranting targeted clinical trials and real-world studies in this specific demographic.

Finally, this case underscores the necessity of SDM. Despite cognitive impairment, the patient articulated a consistent desire for active treatment. Recognizing that processing complex risk-benefit profiles was challenging for him, his family was integrated to support his decision-making. This collaborative approach respected his autonomy and values while aligning the treatment plan with medical realities, exemplifying best practices in geriatric oncology [10,11,20].

## 4. Conclusions

Chemotherapy-free regimens, such as Pola-R, provide a critical therapeutic avenue for vulnerable older patients with DLBCL who are unsuitable for standard cytotoxic therapies. Because clinical evidence in this frail population remains limited, objectively evaluating physiologic reserve through GA is essential. Coupling GA with SDM allows clinicians to safely deploy individualized, chemotherapy-free regimens like Pola-R, maximizing clinical benefit while honoring patient goals and autonomy.

## Figures and Tables

**Table 1:** Comprehensive Geriatric Assessment Findings.

Domain	Assessment Tool	Score/Finding	Clinical Interpretation
Screen.	G8 Score	14/17 (on first admission), 13/17 (at Re-Relapse)	Deductions primarily due to cognitive impairment and reduced mobility
Cognition	Mini-Cog	1/5 (Clock drawing: 0/2, Recall: 1/3)	Mild-to-moderate cognitive impairment, likely related to prior brain irradiation and hydrocephalus
Cognition	MMSE	22/30	
Function	Katz ADL	2/6	Independent only in feeding and continence
Function	Lawton IADL	2/8	Independent only in telephone use and transportation
Comorbidity	CCI	1	Score attributed to dementia/cognitive impairment
Comorbidity	CIRS-G	≥2 in five organ domains	Moderate impairment across multiple organ systems

Note: ADL, activities of daily living; IADL, instrumental activities of daily living; MMSE, Mini-Mental State Examination; CCI, Charlson Comorbidity Index; CIRS-G, Cumulative Illness Rating Scale for Geriatrics.

## Data Availability

The original data presented in the study are included in the article; further inquiries can be directed to the corresponding author.

## References

[1] Ayers E.C., Smith S.M. (2025). Diffuse Large B-Cell Lymphoma in the Older and Frail Patient. Cancers.

[2] Iyengar V., Hamlin P., Torka P. (2025). SOHO State of the Art Updates and Next Questions|Diffuse Large B-Cell Lymphoma in Older Adults: A Comprehensive Review. Clin. Lymphoma Myeloma Leuk..

[3] Lugtenburg P.J., Mutsaers P.G.N.J. (2023). How I Treat Older Patients with DLBCL in the Frontline Setting. Blood.

[4] Dotan E., Chapman A., Kamath C.G. (2021). NCCN Clinical Practice Guidelines in Oncology: Older Adult Oncology. J. Natl. Compr. Cancer Netw..

[5] Loh K.P., Liposits G., Arora S.P., Neuendorff N.R., Gomes F., Krok-Schoen J.L., Amaral T., Mariamidze E., Biganzoli L., Brain E. (2024). Adequate assessment yields appropriate care-the role of geriatric assessment and management in older adults with cancer: A position paper from the ESMO/SIOG Cancer in the Elderly Working Group. ESMO Open.

[6] Dale W., Klepin H.D., Williams G.R., Alibhai S.M.H., Bergerot C., Brintzenhofeszoc K., Hopkins J.O., Jhawer M.P., Katheria V., Loh K.P. (2023). Practical Assessment and Management of Vulnerabilities in Older Patients Receiving Systemic Cancer Therapy: ASCO Guideline Update. J. Clin. Oncol..

[7] Tucci A., Martelli M., Rigacci L., Riccomagno P., Cabras M.G., Salvi F., Stelitano C., Pinotti G., Merli F. (2015). Comprehensive geriatric assessment is an essential tool to support treatment decisions in elderly patients with diffuse large B-cell lymphoma: A prospective multicenter evaluation in 173 patients by the Lymphoma Italian Foundation (FIL). Leuk. Lymphoma.

[8] Merli F., Luminari S., Tucci A., Arcari A., Rigacci L., Hawkes E., Chiattone C.S., Balzarotti M., Spina M., Puccini B. (2021). Simplified Geriatric Assessment in Older Patients With Diffuse Large B-Cell Lymphoma: The Prospective Elderly Project of the Fondazione Italiana Linfomi. J. Clin. Oncol..

[9] Yun X., Bai J., Feng R., Li J., Wang T., Yang Y., Yin J., Qian L., Zhang S., Cao Q. (2024). Validation and modification of simplified Geriatric Assessment and Elderly Prognostic Index: Effective tools for older patients with diffuse large B-cell lymphoma. Cancer Med..

[10] Rostoft S., van den Bos F., Pedersen R., Hamaker M.E. (2021). Shared Decision-Making in Older Patients with Cancer—What Does the Patient Want?. J. Geriatr. Oncol..

[11] Gans E.A., Pieterse A.H., Klapwijk M.S., van Stiphout F., van Steenbergen I.J., Portielje J.E.A., de Groot J.F., van Munster B.C., van den Bos F. (2024). Shared decision-making with older adults with cancer: Adaptation of a model through literature review and expert opinion. Psychooncology.

[12] Sehn L.H., Herrera A.F., Flowers C.R., Kamdar M.K., McMillan A., Hertzberg M., Assouline S., Kim T.M., Kim W.S., Ozcan M. (2020). Polatuzumab Vedotin in Relapsed or Refractory Diffuse Large B-Cell Lymphoma. J. Clin. Oncol..

[13] Coiffier B., Haioun C., Ketterer N., Engert A., Tilly H., Ma D., Johnson P., Lister A., Feuring-Buske M., Radford J.A. (1998). Rituximab (anti-CD20 monoclonal antibody) for the treatment of patients with relapsing or refractory aggressive lymphoma: A multicenter phase II study. Blood.

[14] Tobinai K., Igarashi T., Itoh K., Kobayashi Y., Taniwaki M., Ogura M., Kinoshita T., Hotta T., Aikawa K., Tsushita K. (2004). Japanese multicenter phase II and pharmacokinetic study of rituximab in relapsed or refractory patients with aggressive B-cell lymphoma. Ann. Oncol..

[15] Chavez J.C., Bastos-Oreiro M., Diefenbach C., Lossos I.S., Shah N., Assouline S., Olszewski A.J., Naik S., Ghosh N., Pham S. (2026). A Randomized Phase II Study of Subcutaneous Mosunetuzumab in Combination With Polatuzumab Vedotin Compared With Rituximab Plus Polatuzumab Vedotin in Patients With Relapsed or Refractory Large B-Cell Lymphoma. Am. J. Hematol..

[16] Russler-Germain D.A., Cliff E.R.S., Bartlett N.L. (2023). Cell-of-origin effect of polatuzumab vedotin in diffuse large B-cell lymphoma. Blood.

[17] Schmitz R., Wright G.W., Huang D.W., Johnson C.A., Phelan J.D., Wang J.Q., Roulland S., Kasbekar M., Young R.M., Shaffer A.L. (2018). Genetics and Pathogenesis of Diffuse Large B-Cell Lymphoma. N. Engl. J. Med..

[18] Bertoni F., Montesinos-Rongen M. (2022). Primary diffuse large B-cell lymphoma of the central nervous system: Molecular and biological features of neoplastic cells. Ann. Lymphoma.

[19] Budde L.E., Zhang H., Kim W.S., Maruyama D., Rego E.M., Norasetthada L., Hong H., Ozcan M., Jeon Y.W., Leão Cordeiro de Farias D. (2025). Mosunetuzumab Plus Polatuzumab Vedotin in Transplant-Ineligible Refractory/Relapsed Large B-Cell Lymphoma: Primary Results of the Phase III SUNMO Trial. J. Clin. Oncol..

[20] Appelbaum P.S. (2007). Clinical Practice. Assessment of Patients’ Competence to Consent to Treatment. N. Engl. J. Med..

